# Supplementary material for: Simulated microgravity induces aging phenotypes in human organoids
Source: bioRxiv. 2026 Oct 1:2026.09.30.755771. Preprint. [Version 1] doi: 10.64898/2026.09.30.755771 (PMC13648711; doi:10.64898/2026.09.30.755771)
Supplement: Supplementary file 1 [file media-1.pdf]

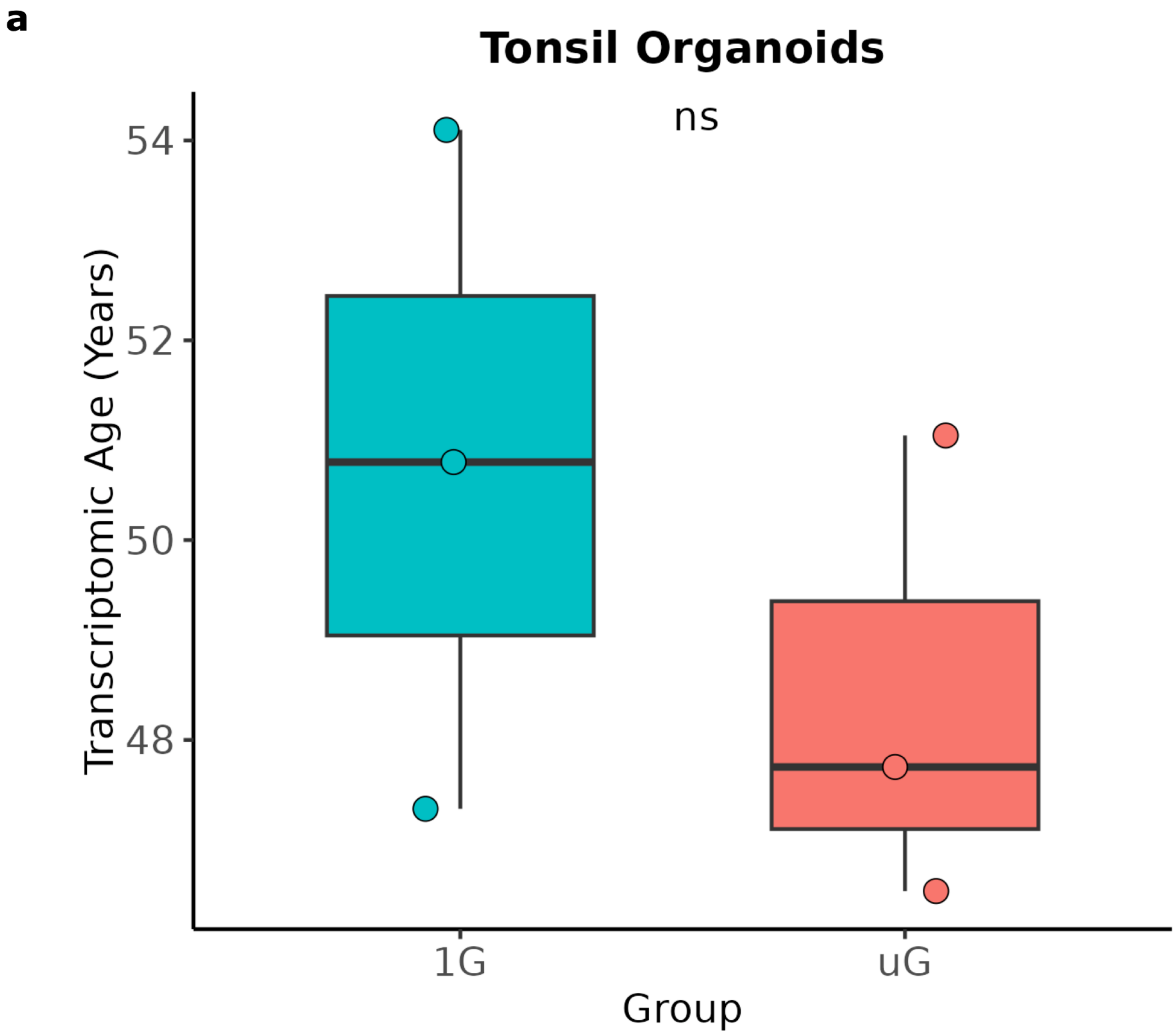

Supplemental Figure 1 | Transcriptomic aging-signature score in tonsil organoids. a, Transcriptomic aging-signature score derived from tonsil organoids generated from younger and older donors in 1g and simulated-microgravity conditions. Points represent individual samples; boxes show the sample distributions. The difference was not significant (ns).
